# Systemic Inhibition of NF-κB Activation Protects from Silicosis

**DOI:** 10.1371/journal.pone.0005689

**Published:** 2009-05-25

**Authors:** Michelangelo Di Giuseppe, Federica Gambelli, Gary W. Hoyle, Giuseppe Lungarella, Sean M. Studer, Thomas Richards, Sam Yousem, Ken McCurry, James Dauber, Naftali Kaminski, George Leikauf, Luis A. Ortiz

**Affiliations:** 1 Division of Occupational and Environmental Medicine, Department of Environmental and Occupational Health, University of Pittsburgh, Pittsburgh, Pennsylvania, United States of America; 2 Department of Environmental and Occupational Health Sciences, School of Public Health and Informational Sciences, University of Louisville, Louisville, Kentucky, United States of America; 3 Department of Physiopathology, University of Siena, Siena, Italy; 4 Simmons Center for Interstitial Lung Disease, Division of Pulmonary, Allergy, and Critical Care Medicine, Department of Medicine, University of Pittsburgh Medical Center, Pittsburgh, Pennsylvania, United States of America; 5 Department of Pathology, University of Pittsburgh Medical Center, Pittsburgh, Pennsylvania, United States of America; 6 Department of Thoracic Surgery, University of Pittsburgh Medical Center, Pittsburgh, Pennsylvania, United States of America; National Heart and Lung Institute, Imperial College London, United Kingdom

## Abstract

**Background:**

Silicosis is a complex lung disease for which no successful treatment is available and therefore lung transplantation is a potential alternative. Tumor necrosis factor alpha (TNFα) plays a central role in the pathogenesis of silicosis. TNFα signaling is mediated by the transcription factor, Nuclear Factor (NF)-κB, which regulates genes controlling several physiological processes including the innate immune responses, cell death, and inflammation. Therefore, inhibition of NF-κB activation represents a potential therapeutic strategy for silicosis.

**Methods/Findings:**

In the present work we evaluated the lung transplant database (May 1986–July 2007) at the University of Pittsburgh to study the efficacy of lung transplantation in patients with silicosis (n = 11). We contrasted the overall survival and rate of graft rejection in these patients to that of patients with idiopathic pulmonary fibrosis (IPF, n = 79) that was selected as a control group because survival benefit of lung transplantation has been identified for these patients. At the time of lung transplantation, we found the lungs of silica-exposed subjects to contain multiple foci of inflammatory cells and silicotic nodules with proximal TNFα expressing macrophage and NF-κB activation in epithelial cells. Patients with silicosis had poor survival (median survival 2.4 yr; confidence interval (CI): 0.16–7.88 yr) compared to IPF patients (5.3 yr; CI: 2.8–15 yr; p = 0.07), and experienced early rejection of their lung grafts (0.9 yr; CI: 0.22–0.9 yr) following lung transplantation (2.4 yr; CI:1.5–3.6 yr; p<0.05). Using a mouse experimental model in which the endotracheal instillation of silica reproduces the silica-induced lung injury observed in humans we found that systemic inhibition of NF-κB activation with a pharmacologic inhibitor (BAY 11-7085) of IκBα phosphorylation decreased silica-induced inflammation and collagen deposition. In contrast, transgenic mice expressing a dominant negative IκBα mutant protein under the control of epithelial cell specific promoters demonstrate enhanced apoptosis and collagen deposition in their lungs in response to silica.

**Conclusions:**

Although limited by its size, our data support that patients with silicosis appear to have poor outcome following lung transplantation. Experimental data indicate that while the systemic inhibition of NF-κB protects from silica-induced lung injury, epithelial cell specific NF-κB inhibition appears to aggravate the outcome of experimental silicosis.

## Introduction

Chronic occupational or environmental exposure to silica is associated with the development of silicosis, a lung disease characterized by granulomatous inflammation and pulmonary fibrosis [1]. In spite of significant progress in its prevention, silicosis remains a major global health problem associated with a high morbidity and mortality for which no specific therapy is available [1]. Silica-induced inflammation is a complex process in which the interaction of silica particles with lung cells is followed by the release of inflammatory mediators [2]. Among these mediators, tumor necrosis factor alpha (TNFα) plays a fundamental role in the pathogenesis of silica-induced lung injury. Mice exposed to silica demonstrate enhanced TNFα production in their lungs in a manner that precedes the inflammatory response and the accumulation of lung collagen [3].

NF-κB is a transcription factor that plays a fundamental role in inflammation [4]–[6]. NF-κB is a protein complex formed from the homo or heterodimers of any of the five members of the rel transcription factor family [REL (c-Rel), RELA (p65), RELB (Rel B), NFKB1 (p50/p105), and NFKB2 (p52/p100)] [4]–[6]. Under basal conditions NF-κB is bound in the cell cytoplasm to IκB, a group [NFKBIA (IκBα), NFKBIB (IκBβ), and NFKBIE (IκBε) are the key members) of NF-κB sensitive proteins that limits NF-κB nuclear translocation and inhibits its ability to bind the promoter region of sensitive genes [6]. NF-κB activation can occur in several pathways: most commonly, it is triggered in a canonical manner in response to inflammatory cytokines (such as TNFα), engagement of T cell receptor, or lypopolysaccharide (LPS) that induces rapid phosphorylation, at Ser32 and Ser36 residues, of IκB by the IκB kinase complex [consisting of the catalytic subunits CHUK (IKKα) and IKBKB (IKKβ)] and several copies of the regulatory subunit called NF-κB essential modifier. Phosphorylated IκBα undergoes ubiquitin-induced degradation by the 26S proteosome and allows the nuclear translocation of the NF-κB, NFKB1-RELA dimers [4]–[6]. A subset of NF-κB activating stimuli such as stimulation of CD40 activate the non-canonical, or alternative, pathway in which activation of catalytic subunits CHUK results in the formation of NFKB2 (p52) from p100 and the generation of NFKB2-RELB heterodimers that target distinct κB elements [4]–[6]. Activation of this alternative pathway of NF-κB is not associated with formation of NFKB1 (p50) dimers [6].

Following inhalation into the lower respiratory tract silica particles interact with epithelial cells and macrophages inducing NF-κB activation [7]–[9]. Binding of NF-κB to DNA promotes the transcription of genes involved in mediating the inflammatory (TNFα) and fibrotic (collagens) responses to silica in mice [10]–[12]. Because of the potential importance of NF-κB in the pathogenesis of silicosis, this transcription factor has been considered a primary target to antagonize silica-induced inflammation in the lung [10], [11], [13]. However, the long-term biologic effects of inhibiting NF-κB in vivo in response to silica are unknown.

Due to the lack of treatment for silicosis, lung transplantation is considered the only a therapeutic alternative. In the present work we characterized the outcome of patients that received lung transplantation for the treatment of their silica-induced respiratory failure. We also treated silica exposed C57BL/6 mice with a pharmacologic inhibitor (BAY 11-7085) of NFKBIA (IκBα) phosphorylation [14] or exposed transgenic mice expressing a dominant negative NFKBIA (IκBα) mutant protein [15], [16] under the control of epithelial specific promoters mice to silica to determine whether the systemic or epithelial inhibition of NF-κB activation is a valid therapeutic approach in silicosis.

## Methods

### Study population

The University of Pittsburgh Institutional Review Board approved the procedures used in this study and information was minimized to comply with HIPPA regulations. We identified 990 patients that received lung transplantation at the University of Pittsburgh during the period of May 1986 to July 2007. From this database we selected patients that received lung transplantation for the diagnosis of silicosis (n = 13) and assessed the overall survival and rate of graft rejection, by measuring the development of bronchiolitis obliterans (BO). We compared the rate of rejection to that of patients with idiopathic pulmonary fibrosis (IPF) that was selected as a control group because survival benefit of lung transplantation has been identified for these patients [17]. Inclusion criteria for IPF included histological evidence of usual interstitial pneumonia (UIP) in the explanted lungs and fulfilled the criteria established by the American Thoracic Society and European Respiratory Society [18], [19]. Diagnosis of silicosis or UIP was unequivocally established by blind re-evaluation (S.Yousem). Upon further evaluation of explanted lungs, two of the twelve patients with diagnosis of silicosis demonstrated evidence of coexistent pneumoconiosis or Kaplan syndrome and were withdrawn for further analysis. Characterization of the TNFα expression and the NF-κB activation was conducted by immunohistochemistry in paraffin-embedded lung tissues (4 µm) isolated from silicosis patients at time of lung transplantation.

### Silica and BAY 11-7085 Treatment

All procedures were approval by the Institutional Animal Care and Use Committee of the University of Pittsburgh. Specific pathogen-free female C57BL/6 (Charles River laboratories, Kingston, NY), and mice genetically deficient in both (p55p75−/−) TNF receptors (The Jackson Laboratory, Bar Harbor, ME) weighing 20 to 25 grams were housed in pathogen-free cabinets as previously described [12], [20]. Animals were exposed to silica (0.2 g/kg) or saline (control) as previously described [12], [20] and killed at selected times (3, 7, 14, and 28 d) after silica exposure. Prior to exposure, crystalline silica (α-quartz, average diameter: 1.7 µm, U.S. Silica Co., Berkeley Springs, WV) was sterilized (200°C, 16 h) to inactivate endotoxin contamination. Silica suspension in sterile 0.9% NaCl (Baxter Healthcare Corp., Deerfield, IL) was prepared by sonicating immediately prior to intratracheal instillation [12], [20]. Groups of mice received 10 mg of BAY 11-7085 (BIOMOL International L.P. Plymouth Meeting, PA)/Kg body weight/day/28 d by i.p. injection in 180 µl of vehicle (0.5% (w/v) methylcellulose in water) or vehicle alone (control). Mice were anesthetized with sodium pentobarbital (200 mg/kg i.p., Henry Schein, Indianapolis, IN) followed by exsanguination via severing of the posterior abdominal aorta. All the lung samples were harvested at a terminal anesthesia point, snap frozen in liquid nitrogen, and stored at −80°C for RNA or hydroxyproline content analysis. Lung hydroxyproline was determined according to the method of Kivirikko et al. as previously described [12], [20].

### Generation of CCSP and SPC-dnIκBα transgenic mice

To inhibit NFKBIA (IκBα) in the airway epithelium, a CCSP-dnIκBα construct was generated by cloning a dominant-negative (dn) IκB mutant [15] under the regulation of rat Clara cell secretory protein (CCSP) promoter [21]. Details of the generation of the CCSP-dnIκBα mice are included in the supplemental information (Method S1). In addition, transgenic SPC-dnIκBα mice (kind gift of Christopher Wilson) were generated at the University of Washington and have dnIκBα under the regulation of the human SPC (SFTPC) promoter as previously described [16]. CCSP- and SPC-dnIκBα mice were crossed with C57BL/6 strain mice for a minimum of 7 generations before used in experiments. Transgene negative littermates were used as controls. Mice were housed under specific-pathogen free conditions in filtered-air cages, and permitted unlimited access to sterile food and water.

### Morphology and Morphometry

Inflammatory cell infiltration was determined by morphometric assessment (conducted blindly by G. Lungarella) and expressed as percentage volume-densities of fibrosis [Vv(f)] was measured according to the stereological principle of Glagoleff and Weibel as previously described [22], [23]. Point counting was performed at a magnification of ×100, by determining 20 random fields per slide and using a multipurpose grid to count 45 points/field to a total of 900 points/specimen. This method measures inflammatory cell infiltration within alveolar septae and alveolar spaces with deposition of extracellular matrix.

### Quantitative real-time polymerase chain reaction (qRT-PCR) assay

To adequately cover the TNFα and NF-κB pathway and targeted cytokines, 40 transcripts selected were analyzed by qRT-PCR. Total RNA was isolated with TRIzol reagent (Invitrogen, Carlsbad, CA) and quantity was assessed by absorbance (NanoDrop, Thermo Scientific, Pittsburgh, PA). RNA (100 ng) was reverse transcribed (High Capacity cDNA Archive Kit, Applied Biosystems Inc., Foster City, CA) and cDNA was PCR amplified with primers and TaqMan Universal PCR Master Mix (Applied Biosystems). Arranged into functional groups, primers (SABiosciences, Frederick, MD) included: *A. TNF pathway group* (19 transcripts): TNF, TNFSF10, TNFSF14, TNFRSF1A, TNFRSF1B, TNFRSF10B, CD27, CD40, LTBR, TNFAIP3, TRAF2, TRAF3, TRADD, TBK1, FADD, CFLAR, RIPK1, RIPK2, and MAP3K1, *B. NFKB pathway group* (9 transcripts): NFKB1, NFKB2. IKBKB, IKBKE, IKBKG, REL, RELA, RELB, and CHUK, and *C. Cytokine group* (12 transcripts mRNAs): CCL2, IL1B, IL6, CSF2, CSF3, IFNG, IRAK1, IRAK2, NLRP2, KAT2B, STAT1, and EGR1 (All abbreviations are current Entrez Gene official symbols and official full names are presented in Table S1). Analysis was performed with an Applied Biosystems 7900HT System (95°C 10 min; 40 cycles 95°C, 15 s; 60°C, 1 min). The expression of each transcript relative to Heat shock protein 90 kDa alpha (cytosolic), class B member 1 (HSP90AB1) transcript level was determined using the 2^−ΔΔC^T method [18] and normalized to strain-matched mice exposed to saline.

### Northern Analysis and Ribonuclease Protected Assays

Analysis of the expression of collagen, type I, alpha 1 (COL1α1), TNFα, and RPS18 (18S) mRNA was conducted as previously described [12], [20], [23]. Metalloproteinase 2 (MMP2), and tissue inhibitor of metalloproteinase 1 (TIMP1) transcript levels were determined by a ribonuclease protection assay (RiboQuant™, Becton, Dickinson and Company, Franklin Lakes, NJ).

### Isolation of Clara and Alveolar Epithelial type II (AEII) cells, and electrophoretic mobility Shift Assay (EMSA), and Western Blot assays

Clara and AEII cells were isolated from the lung of transgenic and wild-type mice as previously described [24], [25]. Complete description of the isolation protocol is included in supplemental data (Methods S1). Nuclear extracts from Clara cells, AEII cells, or the lungs of silica-treated mice were prepared as previously described [23], [26]. Complete description of the EMSA protocol is included in supplemental data. IκB expression was studied by proving proteins with an affinity purified rabbit polyclonal antibodies specific to total or phospho-IκB [PhosphoPlus IκB -alpha (Ser^32^) antibody kit, New England Biolabs, Ipswich, MA].

### Quantification of apoptosis in lung tissues by Terminal deoxynucleotidyl transferase-mediated dUTP nick end labeling (TUNEL)

Apoptotic cells in lung tissues from mice treated with silica or silica plus Bay 11-7085 (n = 5 mice/group) was performed using a TUNEL assay (Travigen Laboratories, Gaithersburg, MD). The mean of the numbers of TUNEL-positive cells was determined using 3 sections/mouse (×400 light microscopy) as previously described [27].

### Statistics

We analyzed both overall survival and rejection-free survival, using the date of transplant as the start date of follow-up. Survival curves were computed using the Kaplan-Meier method. Differences in survival according to primary diagnosis were assessed using the logrank test and the Cox proportional hazards model. Differences between murine strains were analyzed using ANOVA with Fisher's PLSD test for pair-wise comparison (statview 4; Abacus Concept, INC., Berkeley, CA). The differences in transcript levels are expressed as fold change of silica-treated mice compared to saline exposed mice and significant differences were determined by One-way Analysis of Variance with Holm-Sidak all pairwise multiple comparison procedure. Values are expressed as mean±SEM, and p value<0.05 was considered significant.

## Results

### Patients with silicosis have poor prognosis following lung transplantation

**Figure 1:**
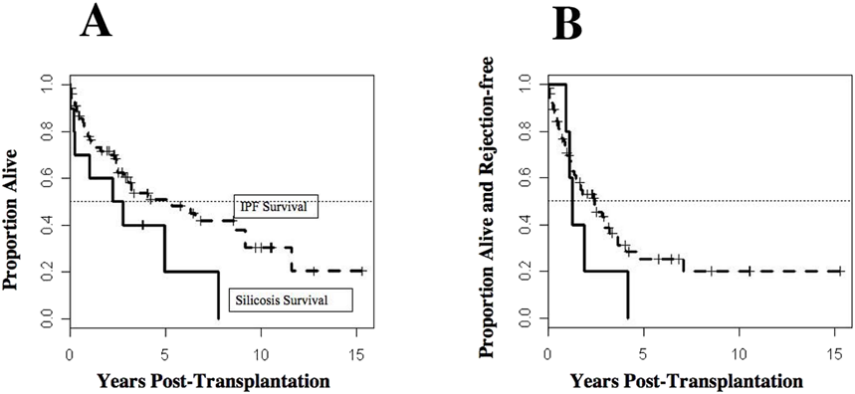
Patients with silicosis have poor outcome following lung transplantation. Figure 1 illustrates the survival rates (1A) and the rejection free time (1B) for patients with idiopathic pulmonary fibrosis (IPF; n = 79) or silicosis (n = 11) following lung transplantation. Survival curves were computed using the Kaplan-Meier method. Differences in survival according to primary diagnosis were assessed using the logrank test and the Cox proportional hazards model as described in the text.

Characteristics of the patients with silicosis and IPF are described in Table 1. We analyzed both overall and rejection-free survival of patients with silicosis or IPF, using the transplant date as the start of follow-up. Ten of the 11 patients with silicosis (average age 52.4±8.6 yr) received single lung transplantation (SLT) and 1 received a double lung transplant (DLT). Of the 79 patients with IPF (average age 54.0±9.9 yr), 71 patients received SLT and 8 patients received DLT. Survival at 30-, 60-, or 90-d did not differ between the two groups as assessed by the Kaplan-Meier method. Differences in survival according to primary diagnosis also were assessed using the log-rank test and the Cox proportional hazards model. Median survival of silicosis patients (2.4 yr, Figure 1A) was somewhat less than that of IPF patients (5.3 yr; lower confidence bounds 0.2: 2.8 yr; p = 0.07 by the logrank test; Figure 1A). This analysis is confounded by gender (i.e., no female silicosis patients) as survival differed between sexes for IPF patients. Thus, female IPF patients were transplanted at a significantly younger age (51.5±9 years) than their male counterpart (55.9±10.2 yr) and demonstrated a significant (p<0.005) survival difference (11.6 yr) when compared to male patients with IPF (3.5 yr). All 11 patients who received lung transplantation for silicosis died during the follow up, while only 37 deaths occurred among the 79 patients with IPF during follow-up. Transplantation type, SLT versus DLT, did not affect survival outcome.

**Table 1:** Patient Characteristics.

	IPF	(n = 79)	Silicosis	(n = 11)	(p-value)
	No. of Patients	%	No. of Patients	%	
**Sex**					
**Male**	53	67.1	11	100.0	
**Female**	26	32.9	0	0.0	(p = 0.03)
**Race**					
**Caucasian**	73	92.4	11	100.0	
**African-American**	5	6.3	0	0.0	
**Hispanic**	1	1.3	0	0.0	(p = 1.0)
**Age at Transplant (years)**					
**Mean**	54.5		52.4		
**SD**	10.3		8.6		
**N**	79		11		(p = 0.31)
**Pre-transplant FVC (L)**					
**Mean**	1.9		1.9		
**SD**	0.69		0.77		
**N**	74		10		(p = 0.76)
**Pre-transplant 6MWD (m)**					
**Mean**	1157.0		993.2		
**SD**	293.6		300.3		
**N**	63		10		(p = 0.13)

P-value denotes the statistical difference between Patients with IPF and those with silicosis.

Rejection of the transplanted lung was defined as the development of bronchiolitis obliterans syndrome (BOS). BOS was defined by an irreversible (≥20%) decrease in forced expiratory volume in one second (FEV1) using each patients' best post transplant FEV1 after lung transplant as baseline, and the development of obliterative bronchiolitis (OB) in lung biopsy of the transplanted lung [28]. The rate of graft rejection increased in silicosis patients (Figure 1B) as compared to IPF patients (p<0.05, by the logrank test). Median graft survival was 0.9 and 2.4 yr, respectively, for the silicosis and IPF groups. A lower confidence bound for the silicosis group was 0.22 yr; a 95% confidence interval for the IPF group was 1.5: 3.6 yr.

### Enhanced macrophage TNFα expression and epithelial NF-κB activation in silicosis

**Figure 2:**
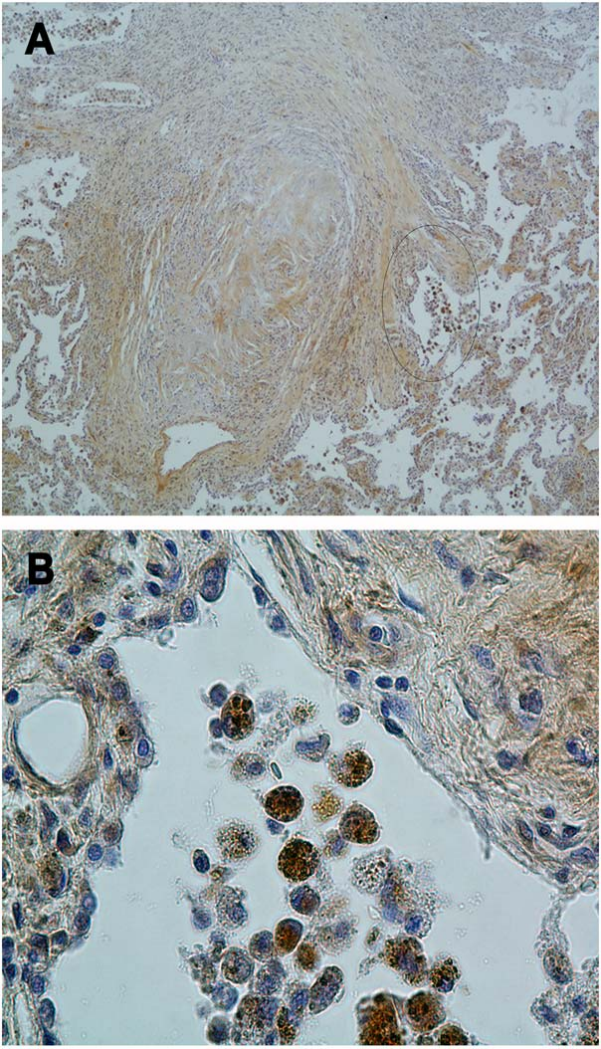
TNFα expression in silicotic lung is predominantly identified in macrophages. Panel A show low photomicrograph (×200) of lung tissue isolated from a silica exposed subject at the time of lung transplantation. Lung parenchyma is replaced by silicotic nodule characterized by a dense palisade of fibroblast surrounding a central fibrinous core that is devoided of cells and abuts the distal airway. Alveolar macrophages accumulate in relatively normal alveolar areas adjacent to the silicotic nodule (circle). Panel B show high (×400) magnification of the area identified in panel A, illustrating immunochemistry signal with anti-TNFα antibody that is confined to cells with macrophage morphology.

TNFα expression and NF-κB activation was determined by performing immune staining in paraffin embedded sections of lung tissues isolated from the lungs of subjects afflicted with silicosis at the time of lung transplantation. In all 11 silicosis patients, large areas of the lung parenchyma were replaced by coalescence of silicotic nodules (Figure 2A). These nodules contain fibrinous material, had few cells, and did not demonstrate TNFα immunostaining (Figure 2A). In contrast, TNFα immunostaining was evident in preserved regions of the lung parenchyma adjacent to the silicotic nodules, which was confined to primarily to macrophages (Figure 2B), and was particularly strong in macrophages that exhibit excessive silica particles in phagosomes (Figure S1). Immunostaining for NF-κB activation was predominantly detected in the nuclei of epithelial cells adjacent to TNFα immunostaining macrophages (Figure S2).

### Lung Transcripts for TNFα, NF-κB, and cytokines increased in silica-treated mice

**Figure 3:**
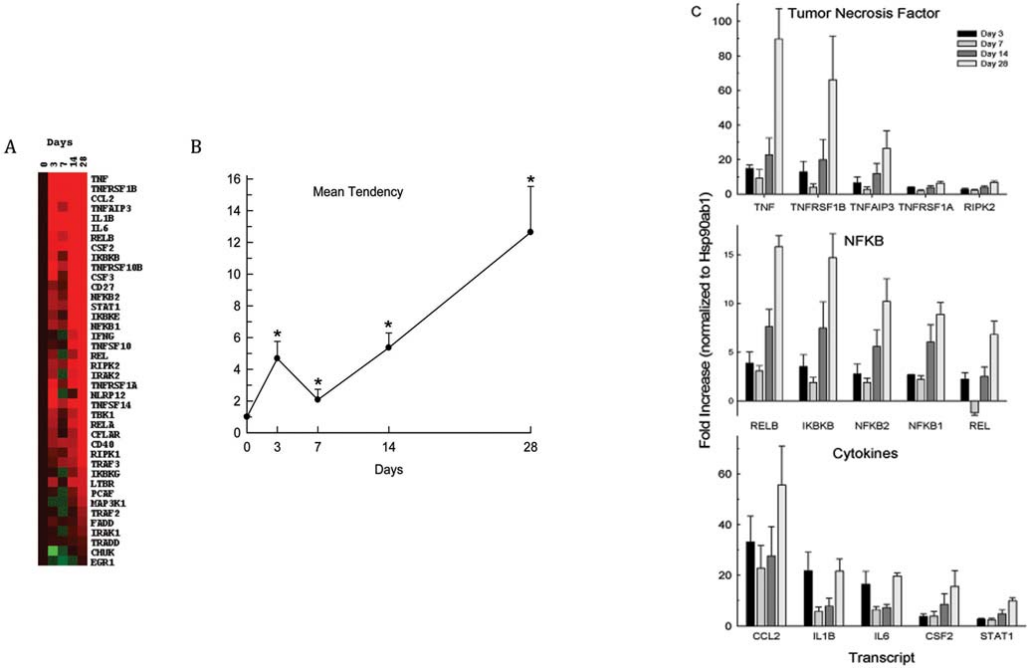
Tumor necrosis factor alpha (TNFα), nuclear factor kappa B (NFKB) and associated cytokines increased in mouse lung following silica treatment. Mice (n = 5 C57BL/6J mice/group) were treated with silica, lung mRNA was obtained at 3, 7, 14, and 28 days, and 40 transcript levels were assessed by qRT-PCR. (A) Self-organizing map visualization of transcripts in Tree View. The order of the transcripts is greatest (top) to lowest (bottom) as measured on day 28. (B) Visualization of Mean Tendency of the 40 transcripts measured. Values are mean±standard error and *p<0.05 as determined by One way analysis of variance with all pairwise multiple comparison procedures (Holm-Sidak method). (C) Transcript levels of selected members of TNFα signaling, NFKB signaling, and downstream cytokine families. All abbreviations are current Entrez Gene official symbols and official full names are presented in Table S1.

To ascertain whether TNFα or NF-κB signaling pathways and targeted cytokines were altered in silica treated mice, 40 lung transcripts were assessed in the lung of C57BL/6 mice at 3, 7, 14 and 28 d after silica exposure. Overall, 26 transcripts increased (p<0.05) within 3 d and were slightly higher than the mean level at 7 d (Figure 3A and Figure 3B). At 28 d, 38 of the 40 transcripts were increased (only CHUK and EGR1 were not significantly increased). Of the 40 transcripts measured, TNFα increased the most (∼90-fold). TNFα receptors, TNFRSF1B and TNFRSF1A, increased, as did transcripts that are rapidly induced by TNFα, TNFAIP3 and RIPK2 (Figure 3C). All 19 TNF signaling pathway transcripts increased by 28 d (Figure 3A). Albeit somewhat less the TNF signaling pathway, NF-κB signaling pathway transcripts also increased in mouse lung following silica exposure. At 28 d, NFKB1 (p50) increased ∼5–10 fold and Rel/NFKB family transcription factors (REL, RELA, RELB) increased ∼5–15 fold (Figure 3C). Only 1 (CHUK) of 9 transcripts in the NF-κB signaling pathway was not significantly different from control by 28 d. In addition, 11 of 12 cytokine transcripts increased significantly with the progression of treatment. Of these, cytokine controlling the production, differentiation, and function of monocytes/macrophages (CCL2 a.k.a. MCP1, and CSF2 a.k.a. GM-CSF), and of granulocytes (CSF2: a.k.a. G-CSF; CCL2) are noteworthy (Figure 3C). In addition, transcripts encoding interleukins (IL1B, IL6), IFNG, and the downstream transcription activator, STAT1, increased markedly (∼8–20 fold at 28 d).

### TNFα receptors mediate NF-κB activation in experimental silicosis

**Figure 4:**
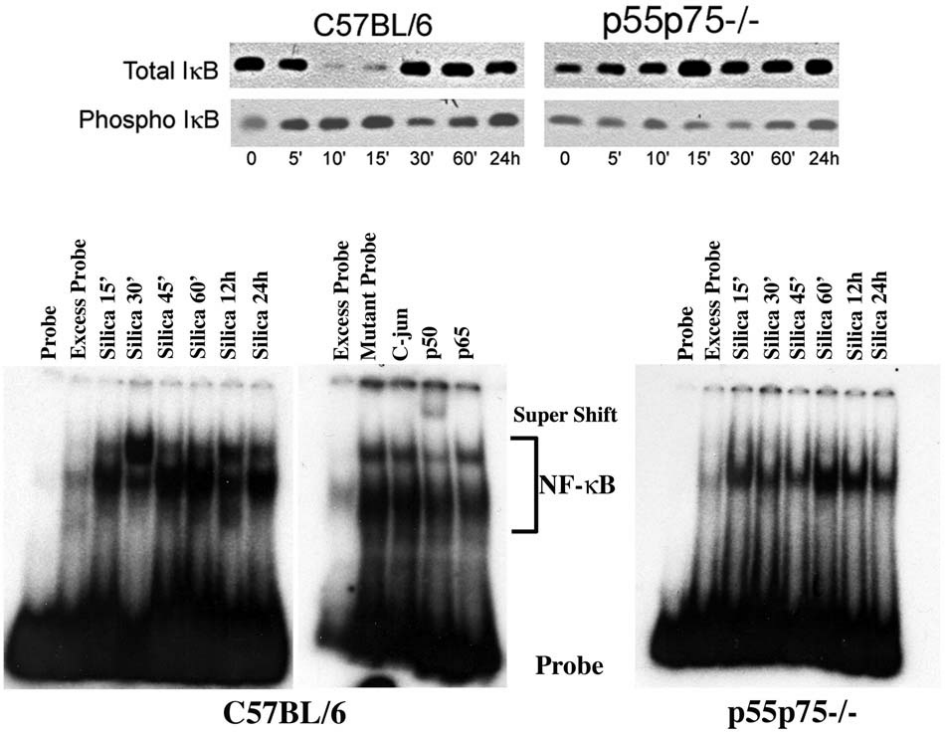
TNFα receptors mediate NF-κB activation in experimental silicosis. Western blot (upper panel) representing expression of total and phosphorylated IκB (phospho IκB) in the lungs of silica-sensitive C57BL/6, or double (p55/p75−/−) TNFα receptor deficient mice following the intratracheal instillation of silica particles as described in the text. Lower panel illustrates the DNA binding activity of NF-κB in crude nuclear extracts from whole lung isolated from C57BL/6, and double (p55/p75−/−) TNFα receptor deficient mice after silica exposure. Silica induces two bands: a lower band representing the p50-p50 homo-dimer, present in both the C57BL/6 and the double (p55p75−/−) TNFα receptor deficient mice, and an upper band, only observed in the lungs of C57BL/6 mice, representing the p50-p60 heterodimer. Excess probe represents NF-κB binding in lung nuclear extract of a silica-treated mouse, assayed in the presence of excess unlabelled oligonucleotide as a competitor. Competition assays were performed using 400 times excess of unlabeled probe or NF-κB mutant oligonucleotide (Santa Cruz). Supershifts were performed by adding to the binding mixture antibodies to p50 (sc1190X) or to p65 (sc372X) or against C-Jun/AP-1 (sc44X) (Santa Cruz) before adding the labeled probe to the mixture.

We have previously reported that TNFα receptors play a fundamental role in experimental silicosis and TNFα receptor deficient mice are protected from silica-induced lung fibrosis [12], [20], [21]. Here we show that TNFα-receptor mediated signaling is an important determinant of the NF-κB activation in the mouse lung in response to silica. Instillation of silica particles increased phophorylated IκB moiety (Figure 4). This increase was followed by IκB rapid degradation and eventual disappearance of (total IκB) in the lungs of silica-sensitive C57BL/6 mice. In contrast to C57BL/6 mice, these changes in IκB did not take place in the lungs of double (p55p75) TNFα receptor deficient mice developed in the same silica-sensitive C57BL/6 genetic background. Silica-induced degradation of phospho IκB is associated with NF-κB activation in the lungs of C57BL/6 (Figure 4). Analysis of NF-κB gel shift in lung nuclear extracts show that silica induces two bands: a lower band representing the p50-p50 (NFKB1-NFKB1) homodimer, present in both the C57BL/6 and the double (p55p75−/−) TNFα receptor deficient mice, and an upper band, only observed in the lungs of C57BL/6 mice, representing the p50-p65 (NFKB1:RELA) heterodimer (Figure 4). Supershift assays confirmed that the lower band could be competed by excess cold, but not by mutant, probe while the upper band could be displaced by incubation of the nuclear extract with an antibody against the p50 or the p65 complex (Figure 4). These data indicate that silica induces a canonical, TNFα receptor-mediated, activation of NF-κB in the mouse lung.

**Figure 5:**
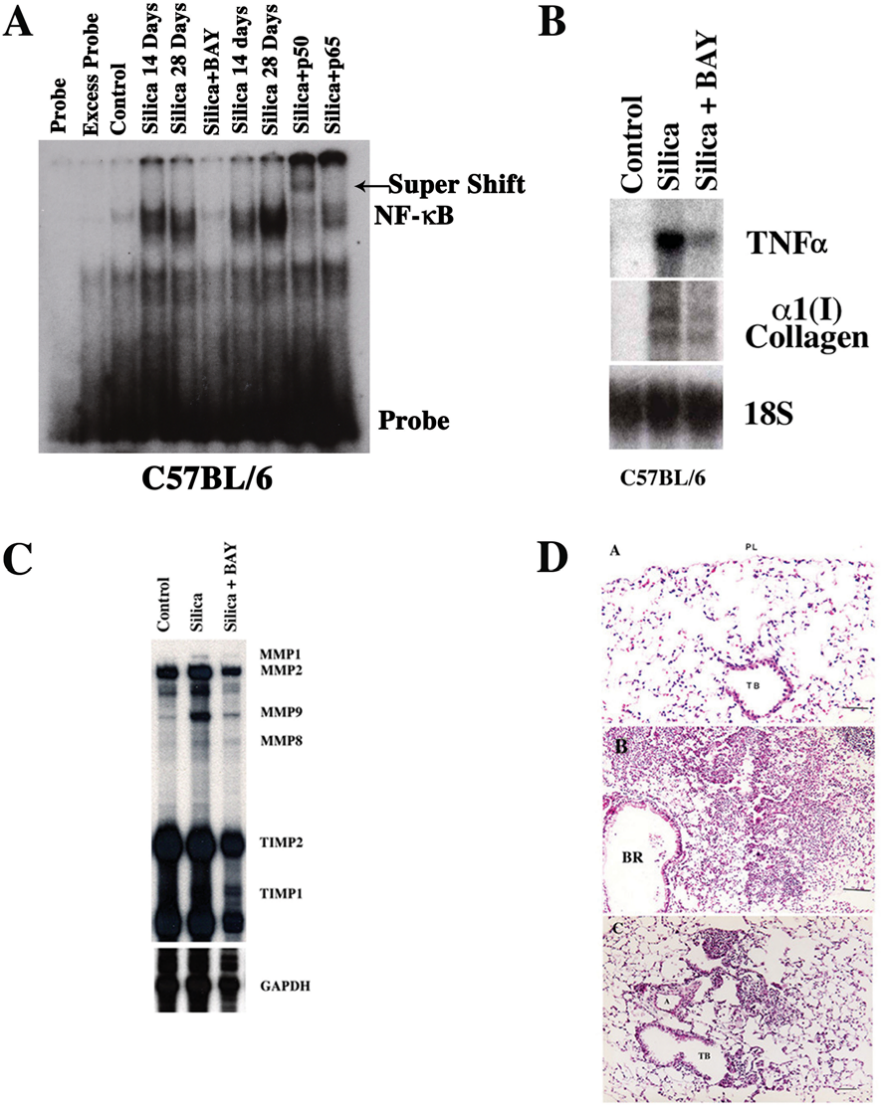
Systemic inhibition of NF-κB activity ameliorates silica-induced lung injury in mice. A) DNA binding activity of NF-κB in crude nuclear extracts from whole lung isolated from C57BL/6 mice 14 and 28 days after silica, or silica+BAY exposure. Excess probe represents NF-κB binding in lung nuclear extract of a silica-treated mouse, assayed in the presence of excess unlabelled oligonucleotide as a competitor. Antibody supershifts were performed using the nuclear extract of a C57BL/6 silica-treated mouse as described in Methods section. B) Northern blot analysis of TNFα, α1(I) collagen, and 18S (loading control) mRNA expression in mouse lung 14 days following the intratracheal injection of saline as control, silica alone, or silica+BAY as described in Methods section. C) The effect of BAY on metalloproteinase (MMPs) RNA expression in mouse lung following silica exposure. Total lung RNA was isolated from the lungs of C57BL/6 mice 14 days after control, silica, or silica+BAY and subjected to RPA as described in Methods section. D) The panels show low (×100) power magnification photomicrographs of the lung obtained from lung tissues of C57BL/6 mice exposed to saline as control (A), silica (B), or silica+BAY (C) as described in the Methods section. TB = terminal bronchiole. Bar = 20 µm. Gel illustrations are representative of 4 different experiments.

Following the early activation of NF-κB, lung IκB mRNA increased in silica-exposed C57BL/6 mice (not shown) and IκB protein returned to baseline (Figure 4). Enhanced NF-κB activation was appreciated in the lungs of silica-exposed C57BL/6 mice for the entire duration of the experiments, and it was readily demonstrated 14 and 28 d after silica exposure (Figure 5). Therefore, it is conceivable that inhibition of NF-κB activity could in turn modulate expression of NF-κB sensitive genes and alters the development of silica-induced lung injury in these mice.

### Systemic inhibition of NF-κB activity ameliorates silica-induced lung injury in mice

To investigate whether inhibiting systemic or lung epithelial cell-specific NF-κB activation is protective in the development of experimental silicosis we adopted two experimental approaches. Systemic inhibition of NF-κB activation was accomplished using a novel pharmacologic agent, BAY-117821, which irreversibly inhibits TNFα-induced phosphorylation of IκB in human endothelial cells, and NF-κB activation in silica stimulated macrophages [14], [23]. In preliminary studies, dose responses (2.5–50 mg/kg) were tested and 10 mg/kg bwt/d (180 µl 0.5% (w/v) methylcellulose i.p.) was capable of consistently inhibiting the silica-induced activation of NF-κB in multiple organs including the lungs of C57BL/6 mice without apparent toxicity to the mice (Figure 5).

Silica treatment induced NF-κB activation, 14 and 28 d, in the lungs of C57BL/6 mice (Figure 5A). Activation of NF-κB was associated with increased inflammatory (TNFα), fibrotic (COL1A1), and metalloproteinases (MMPs) transcripts in the lungs of silica-exposed C57BL/6 mice (Table 2, Figure 3C, Figure 5B&C, and Figure S3). Compared to control-treated mice, silica-exposed C57BL/6 mice developed fibrosis with increased percentage of fibrosis [(Vv(f)] (Table 2, Figure 5D), lung hydroxyproline (Table 2), and apoptotic cells, identified as TUNEL positive cells (Figure S4, and Table 3). Treatment of silica-exposed C57BL/6 mice with BAY 11-7085 compound inhibited the lung NF-κB activation (Figure 5A), inflammatory and fibrotic transcripts (Table 2, Figure 5B&C, and Figures S3), and histological evidence of inflammatory infiltrates, fibrotic lesions (Figure 5D, Table 2), and TUNEL positive cells (Table 3).

**Table 2:** The effect of BAY Treatment on Silica Induced Gene Expression.

Treatment Condition	TNFα	α1(I) Collagen	TIMP-1	MMP1
**C57BL/6 Control**	0.164±0.004	0.310±0.014	0.079±0.06	0.014±0.004
**C57BL/6 silica**	0.215±0.006*	0.440±0.019*	0.10±0.010 *	0.020±0.007*
**C57BL/6 Silica+BAY**	0.172±0.005†	0.340±0.007†	0.088±0.013†	0.014±0.002†

* Statistically significant different when compared to control-treated mice.

† Statistically significant different when compared to silica exposed mice.

**Table 3:** The effect of BAY Treatment on Silica Induced Lung Injury.

Treatment Condition n = 6 mice/group	Hydroxyproline (µg/Left Lung)	Volume-Density Fibrosis Vv (%f)	TUNEL-Positive cells (per mm^2^)
**C57BL/6 Control**	46±5	1±0.1	1±0.6
**C57BL/6 Silica**	87±18*	17±6.4*	64±35*
**C57BL/6 Silica+BAY**	60±12* †	8±0.1* †	25±11* †

* Statistically significant different when compared to control-treated mice.

† Statistically significant different when compared to silica exposed mice.

**Figure 6:**
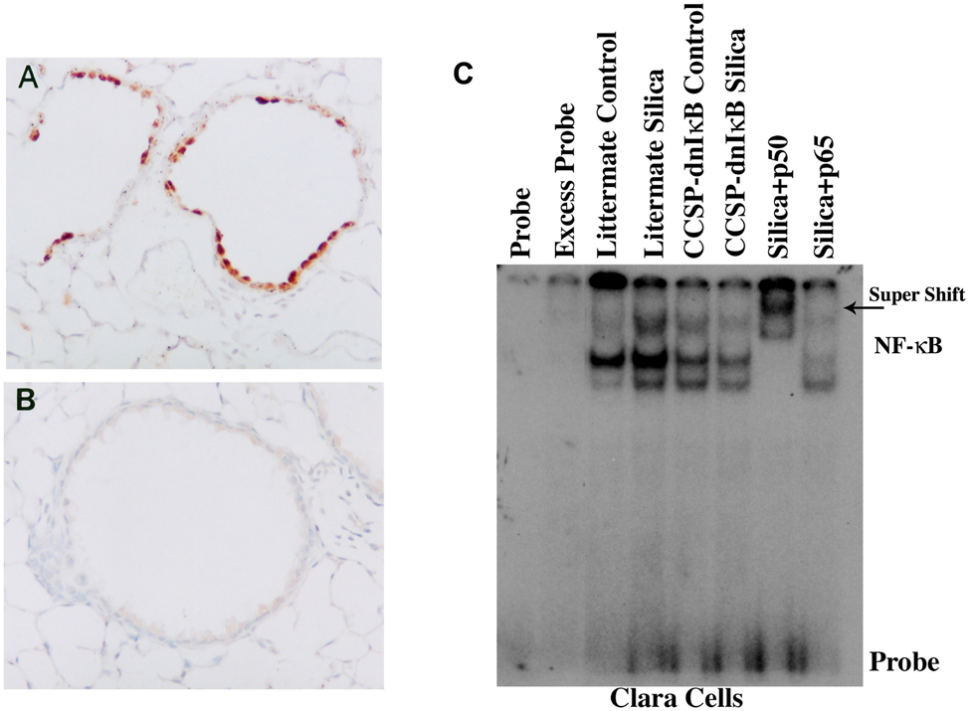
Expression of a dominant-negative (dn) IκBα protein inhibits epithelial activation of NF-κB in response to silica. Lung sections from CCSP-dnIκBα transgenic mice were stained with antibodies against the modified (dnIκBα) IκBα peptide. Panel A shows strong staining in Clara cells of a dnIκBα transgenic mouse. Staining with the same antibody is absent in the non-transgenic littermate mouse in panel B. DNA binding activity of NF-κB in crude nuclear extracts from Clara (C) cells isolated from the lungs of CCSP-dnIκBα transgenic mice, or their littermate (wild-type) 28 days after exposure to silica or saline as controls as described in Methods section. Excess probe represents NF-κB binding in nuclear extract from Clara isolated from silica-exposed littermate wild type mice, assayed in the presence of excess unlabelled oligonucleotide as a competitor. Antibody supershifts were performed using the nuclear extract from Clara cells isolated form silica-exposed littermate wild type mouse as described in Methods section.

To achieve lung epithelial cell-specific inhibition of NF-κB activation, we used mice in which dominant-negative IκBα (dnIκBα:NFKBIA) mutant protein [15] is targeted to epithelial cells under the control of the rat Clara cell secretory protein (CCSP: Scgb1a1) or the human surfactant protein C (SPC:SFTPC) promoter respectively. These dnIκBα mutant mice, the mutant protein is resistant to phosphorylation and proteosomal degradation [15], [16] and inhibit NF-κB activation in those cells in which the transgene is expressed (Figure 6). To confirm that that NF-κB activation was impaired in the epithelium of these mice we isolated Clara or alveolar epithelial type II cells from these transgenic mice and non-transgenic littermate controls following exposure to silica. In vivo exposure (28 d) to silica readily induced NF-κB activation in Clara (Figure 6) or alveolar epithelial type II cells isolated from the littermate wild-type mice (not shown). In contrast, this NF-κB activation is greatly reduced in Clara or alveolar epithelial type II cells isolated from CCSP- or SPC-dnIκBα mice (Figure 6).

**Figure 7:**
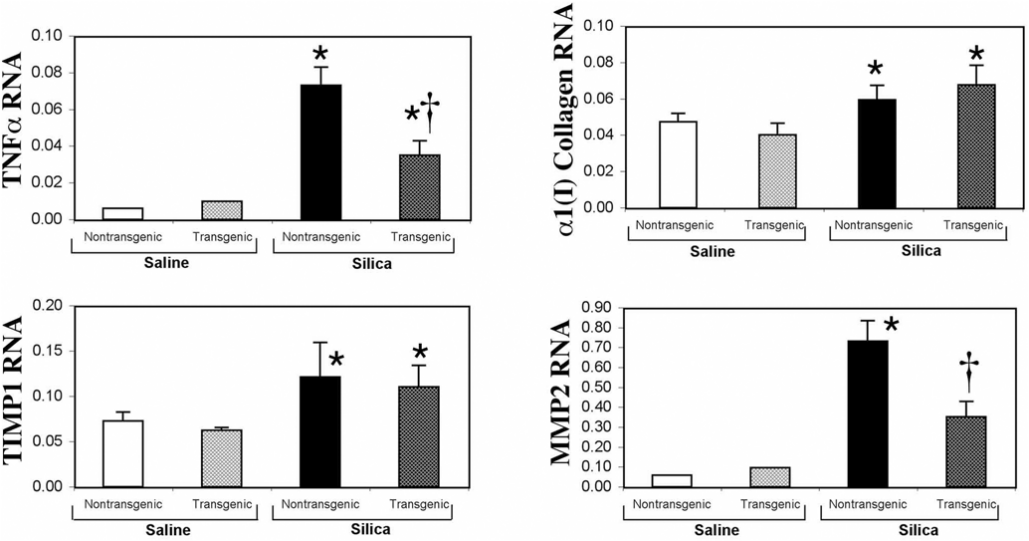
Clara cell expression of a dominant-negative (dn) IκBα protein alters TNFα, and MMP2, but not α1(I) collagen and TIMP-1, mRNA expression in response to silica. Densitometry analysis of Northern blots of TNF, α1(I) collagen, MMP2, TIMP-1, and 18S (loading control) mRNA expression in CCSP-dnIκB mouse lung 28 days following the intratracheal injection of saline as control, or silica as described in the Methods section. Compared to their wild type littermates, CCSP-dnIkB transgenic mice significantly inhibited (P<0.05) the enhanced expression of TNFα and MMP2 mRNA induced by silica in the mouse lung. Figure illustrate results obtained from five set of mice exposed to silica or saline as control. * Indicates statistically significant difference compared to saline treated mice. † Indicates statistically significant difference compared to wild-type non-transgenic mice.

CCSP-dnIκBα mice reacted to silica exposure with decreased lung inflammation as compared to littermate controls (Table 4). This attenuated inflammatory response was associated decreased TNFα transcripts and inflammatory cell infiltration [(Vv(f)] at 28 d after silica exposure (Table 4). In contrast to these effects on inflammation, lung hydroxyproline deposition increased in CCSP-dnIκBα mice than in littermate controls (Table 4). Similarly, CCSP-dnIκBα mice and wild-type littermate mice had nearly equivalent increases in collagen and TIMP1 transcripts. However, silica-induced lung MMP2 transcripts were less in CCSP-dnIκBα mice as compared to C57BL/6 mice (Figure 7). Because NF-κB can function as an anti-apoptotic factor, we evaluated the effect of expressing the dnIκBα mutant in the lung epithelium of these transgenic mice. CCSP-dnIκBα mice had increased apoptosis (TUNEL positive cells) in their lungs when compared to their non-transgenic littermate controls (Table 4). SPC-dnIκBα mice reacted to silica exposure similar to that of CCSP-dnIκBα mice. SPC-dnIκBα mice had decreased infiltrating cells, but accumulated increased hydroxyproline and TUNEL positive cells in their lungs when compared to littermate controls (Table 5).

**Table 4:** Silica Induced Lung Injury in CCSP-dnIKBα mice.

Treatment Condition n = 5 mice/group	Hydroxyproline (µg/Left Lung)	Volume-Density Fibrosis Vv (%f)	TUNEL-Positive cells (per mm^2^)
**NT Littermate Control**	39.7±4.0	4±0.7	0.5
**CCSP-dnIκB Control**	60±2	2±0.5	4
**NT Littermate Silica**	76±3*	12±3*	26±8*
**CCSP-dnIκB Silica**	85±5*	7±0.5* †	46±1* †

* Statistically significant different when compared to control-treated mice.

† Statistically significant different when compared to silica exposed NT littermate mice.

**Table 5:** Silica Induced Lung Injury in SPC-dnIκBα mice.

Treatment Condition n = 6 mice/group	Hydroxyproline (µg/Left Lung)	Volume-Density Fibrosis Vv (%f)	TUNEL-Positive cells (per mm^2^)
**NT Littermate Control**	47±2	0	0
**SPC-dnIκB Control**	51±9	2±0.5	4
**NT Littermate Silica**	97±10*	25±4	27±14
**SPC-dnIκB Silica**	117±7* †	18±4* †	48±18* †

* Statistically significant different when compared to control-treated mice.

† Statistically significant different when compared to silica exposed NT littermate mice.

## Discussion

Silicosis remains a severe lung disease for which no successful treatment is available. Here, we report that silica exposure leads to large portion of the lung replaced by coalescing nodules with proximal TNFα expressing macrophage and NF-κB activation in epithelial cells. We also found that patients with silicosis had poor survival and experienced early rejection of their lung grafts following lung transplantation. Using a mouse experimental model of silicosis in which the endotracheal instillation of silica reproduces the silica-induced inflammation observed in humans we show that systemic inhibition of NF-κB activation with a pharmacologic inhibitor (BAY 11-7085) of IκBα (NFKBIA) phosphorylation significantly decreases silica induced inflammation and fibrosis, with reduced collagen deposition and apoptosis. In contrast, transgenic mice expressing a dominant negative IκBα mutant protein under the control of epithelial specific promoters demonstrate enhanced collagen deposition and apoptosis in their lungs in response to silica.

The worldwide incidence and prevalence of silicosis is uncertain, but is increasing in developing countries [29]. In the United States the incidence and prevalence of silicosis is under-estimated as silicosis is not a reportable disease and under recognized [30]. Approximately 1.7 million people in the US are occupationally exposed to silica [31]–[34] and as many as 119,000 are exposed above permissible levels [31]–[34] with the majority of these individuals employed in mining, construction or maritime activities. Consequently, an estimated 3,600–7,300 newly recognized silicosis cases in the US from 1987 to 1996 [31], [33], [34]. Although preventive measures have significantly decreased the mortality attributable to silica the reality is that that a large number of silica-exposed patients are still dying as a result of this disease [34]. While mortality is widely spread across the country, nine states reported high rates of mortality, more than 2–3 deaths/million people/year, with clusters of counties where mining is an important activity contributing disproportionably (>14.8 deaths/million/year) to this mortality [34]. A global health issue, silicosis levels are rising in developing countries. For example, the silicosis burden in China is growing (with ∼7–10,000 new cases/yr) and is extensive (>500,000 cases resulting in ∼24,000 deaths/yr) [29].

Once established, silica-induced inflammation progresses even after occupational exposure has ended, a phenomenon clearly reproduced in experimental animals [35]. Using conventional anti-inflammatory agents does not control silica-induced inflammation and therefore, because of this lack of successful treatment, lung transplantation has been proposed as an effective therapeutic alternative [1]. However, very little published data is available to support this postulate. A SLT was reported in a 23-yr male in 1972 with the patient surviving 10 months [36]. More recently, the Copenhagen National Lung Transplant Group reported 4 translation cases, but no discussion of their outcome was available [37].

In this report, we describe our experience at the University of Pittsburgh (a post industrial city located in the heart of the mining industry in Western Pennsylvania) with 11 silicosis patients that received lung transplantation for this disease from 1986–2007. The outcome of these patients was contrasted with that of a group of 79 patients that received lung transplantation for IPF, a diffuse parenchymal lung disease for which lung transplantation confers a survival benefit [17]. Our data indicate that patients with silicosis appear to have poor survival and greater rate of graft rejection, despite identical protocols for immunosupression, than patients with IPF following lung transplantation. Our analysis is limited by the small size and more importantly by gender differences in lung transplant survival among patients with IPF. Female IPF patients were transplanted at a younger age and had better survival outcome and lower rejection rates when compared to their male counterparts. The reasons for the gender difference in survival following lung transplantation are unknown and are worthy of additional study.

To further our understanding of the pathogenesis of silicosis we used well-characterized mouse model in which silica exposure produces lung inflammation and parenchymal nodules comparable to those observed in humans exposed to silica. Using this experimental model, silica induced rapid (with in minutes) and persistent (identified 14 and 28 d after a single intratracheal instillation) activation of NF-κB in the lung and that TNFα receptors greatly contribute to this activation. Previously, TNFα has been implicated as a central mediator in the pathogenesis of silicosis. Piguet et al. reported that the exposure of mice to silica increased lung TNFα mRNA levels [3], which preceded the development of lung inflammation and collagen deposition [11], [12]. Furthermore, these investigators were able to abrogate silica-induced inflammation and lung fibrosis with the administration of anti TNFα antibodies or TNFα receptors that antagonized the bioactive TNFα [3], [38]. Similarly, we have previously reported that animals deficient in TNFα receptors are protected from silica-induced inflammation and fibrosis [12], [20]. In humans, compelling evidence indicates that polymorphism of the TNFα gene promoter (TNF-308: denominated TNF-A allele 2) is associated with silicosis disease severity, although not with disease frequency, in South African miners [39].

TNFα mediates its biologic action by binding to two receptors of tumor necrosis factor receptor superfamily, member 1A (TNFRSF1A: p55) and 1B (TNFRSF1B: p75) [40], [41]. In this work we find that TNFα receptors contribute to the canonical activation of NF-κB in the mouse lung in response to silica. Previously, we reported that double TNFα receptor deficient mice failed to activate NF-κB in their lungs in response to silica [12]. Centrally located in the TNFα signal pathway, NF-κB activation mediates the inflammatory and cell survival effects of TNFα and therefore constitutes a logical target for antagonism in silicosis [7]–[13]. The long-term effects of such inhibition are not well understood.

The present work underscores the importance of the specificity of the NF-κB antagonism during lung inflammation. Thus, both the inflammatory and fibrotic responses to silica was ameliorated by systemic inhibition of NF-κB activation with BAY-117821, an irreversibly inhibitor of TNFα-induced phosphorylation of IκB in human endothelial cells [14]. In contrast, although the lung epithelial cell specific inhibition of NF-κB also reduced TNFα expression and the infiltration of inflammatory cells in the lung, it accentuated the apoptotic process and enhanced the fibrosis response by promoting the deposition of collagen and decreasing the matrix remodeling in the lung in response to silica.

These data stress two aspects of the pathophysiology of silicosis. The first aspect is that although silica enters the body via the respiratory system it triggers a systemic response characterized by stimulation of bone marrow and the migration of bone marrow-derived cells into the lung [42]–[45]. Published data in experimental models of lung fibrosis indicate that although this response is dominated by migration of neutrophils and lymphocytes [42]–[45] it also includes the homing of profibrotic fibrocytes [46], [47]. Characterized by their expression of hematopoietic markers (such as CD45) fibrocytes also express collagen and contribute actively to the deposition of this protein in the lung [47]. Mobilization and homing of fibrocytes into the lung is facilitated by their expression of chemokine receptors, such as CXCR4, and the enhanced expression in the injured lung of the cognate ligands for these receptors (i.e., stromal cell derived factor1 alpha and CXCL12). Because expression of these ligands is sensitive to NF-κB regulation [48], early inhibition of the NF-κB activation could reduce the migration of these cells and protect the lung from further injury.

The second aspect is that epithelial cells responses contribute to inflammation in silicosis. Consistent with this statement, CCSP-dnIκB as well as SPC-dnIκB transgenic mice exhibit reduced TNFα mRNA expression and are less inflamed than their wild-type littermates. However, these mice exhibit more robust accumulation of collagen in response to silica. These data underscore the importance of the epithelial specific inhibition of NF-κB, which has previously been reported to increase MMPs in the lungs of silica-exposed animals depleted of Clara cells [49]. In contrast, we find that silica induced expression of the MMP inhibitor, TIMP1, in the lung of mice, and this enhanced expression was not altered in the lungs of the CCSP-dnIκB mice. Therefore, these data suggest that the decrease MMPs expression originated by the Clara cell specific inhibition of NF-κB would create an imbalance of the ability of lung to remodel in favor of matrix deposition. Our data are also consistent with the hypothesis that lung epithelium harbors a small number of stem cells that are resistant to toxins and are fundamental to the maintenance of homeostasis of the lung [50]. Therefore, it is plausible that expression of the IκB repressor under specific epithelial promoters, such as CCSP or SPC, results in reduced activation of NF-κB on these cells rendering them more sensitive to apoptosis thus compromising their ability to repair the injured epithelium.

In summary, the present work re-emphasizes that silicosis remains a serous lung disease for which no specific treatment is successful. Although limited by its size, our data support that patients with silicosis appear to have poor outcome following lung transplantation. Experimental data indicate that TNFα receptors contribute to the silica-induced activation of NF-κB in the lung and our data suggest that early and systemic antagonism of this signal transduction ameliorates lung inflammation and benefits outcome in experimental silicosis. Because systemic antagonism of TNFα in humans is currently possible with a variety of strategies such as use of antibodies or recombinant receptors that bind and antagonize TNFα it would be appropriate to prospectively study the potential therapeutic role of such antagonism in modulating silica-induced lung injury and improving the outcome of lung transplantation for silicosis.

## Supporting Information

Figure S1 Silica exposed Macrophages express TNFα. High (×400) magnification photomicrograph, using polarized light of the same area illustrated in Figure 3B, to illustrate that TNFα expression is predominantly located in macrophages laden with bi-refringent silica particles.
(7.06 MB TIF)

Figure S2 TNFα and NF-κB expression in silicosis. Panel A show photomicrograph (×100) illustrating hematoxyllin and eosin staining of silicotic nodule involving a terminal airway in lung tissue isolated from a silica exposed subject at the time of lung transplantation. Insert (circle) represents an area adjacent to the silicotic nodule to illustrate sequential HE (D ×200), and immunohistochemistry staining against TNFα in interstitial macrophages (arrows) loaded with dust (E ×200), or nuclear localization on NF-κB in adjacent epithelial cells (E ×200).
(5.84 MB TIF)

Figure S3 The Effect of BAY treatment on silica-induced gene expression in the mouse lung. Northern blot analysis of TNF, α1(I) collagen, TIMP-1, and 18S (loading control) mRNA expression in mouse lung 28 days following the intratracheal injection of saline as control, silica alone, or silica+BAY as described in Methods section. Gel is representative of result obtained with three different sets of animal exposures.
(0.34 MB TIF)

Figure S4 The effect of systemic or lung epithelial specific NF-κB inhibition on the induction of apoptosis (TUNEL staining) in the lung of silica exposed mice. Apoptosis was characterized by terminal deoxynucleotidyltransferase dUTP nick end-labeling (TUNEL) as described in Methods section. The panels show low (×100) power magnification photomicrographs of the lung obtained from lung tissues of C57BL/6 mice exposed to saline as control (A), silica (B), silica+BAY (D), or SPC-dnIκB transgenic mouse (E) exposed to silica as described in the Method section. Panel C show high (×400) magnification of TUNEL positive cells identified in B. Panel F illustrates negative staining (by omission of treatment with deoxynucleotidyltransferase enzyme) of a silica-exposed tissue used as control to demonstrate stain specificity.
(10.28 MB TIF)

Table S1 (0.07 MB DOC)

Methods S1 File containing description of supplemental methods
(0.05 MB DOC)
