# Supplementary figures and images for: Systemic Inhibition of NF-κB Activation Protects from Silicosis
Source: PLoS One. 2009 May 25;4(5):e5689. doi: 10.1371/journal.pone.0005689 (PMC2682759; doi:10.1371/journal.pone.0005689)

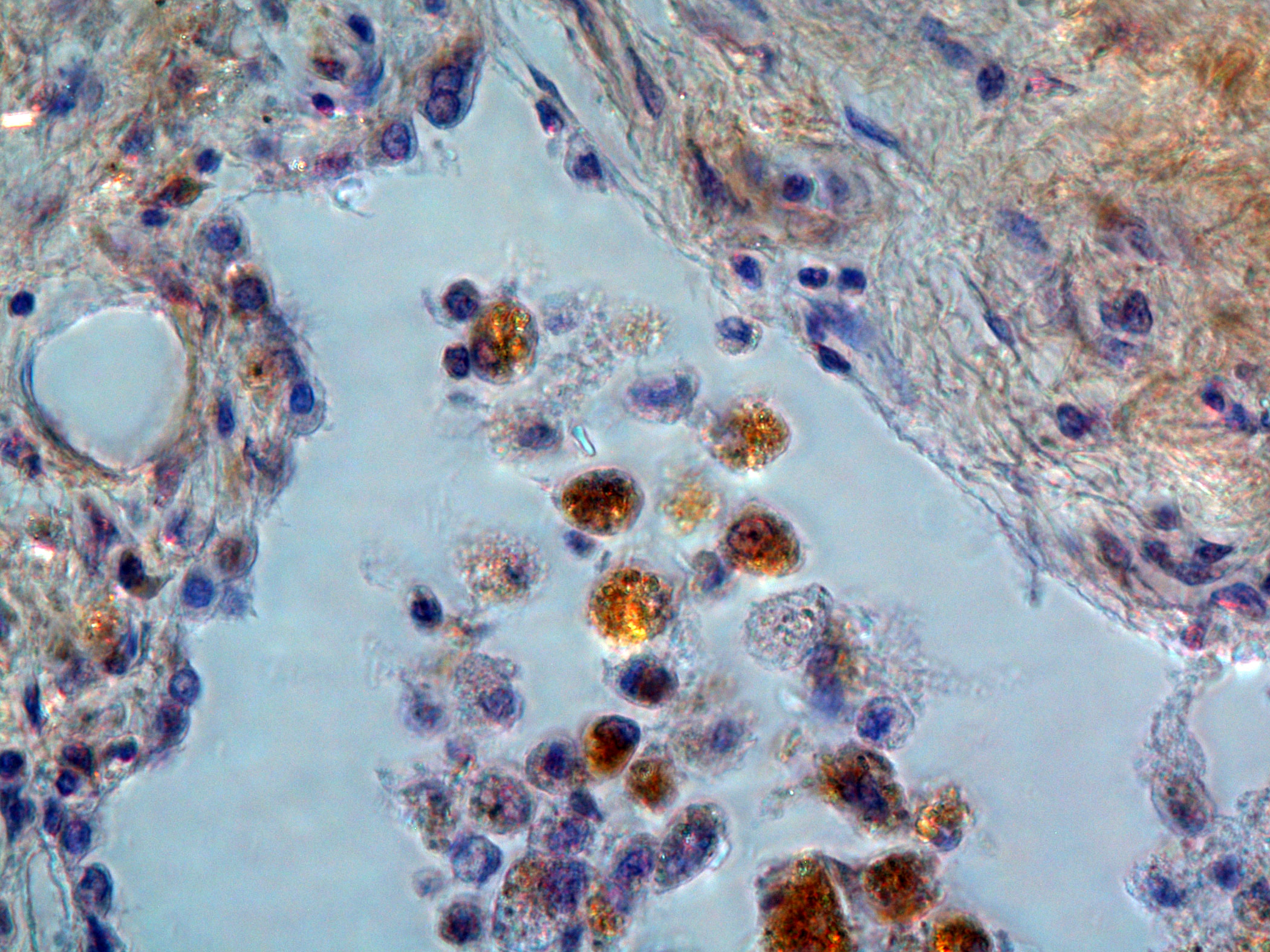

Supplement: Figure S1 — Silica exposed Macrophages express TNFα. High (×400) magnification photomicrograph, using polarized light of the same area illustrated in Figure 3B, to illustrate that TNFα expression is predominantly located in macrophages laden with bi-refringent silica particles. (7.06 MB TIF) [file pone.0005689.s001.tif]

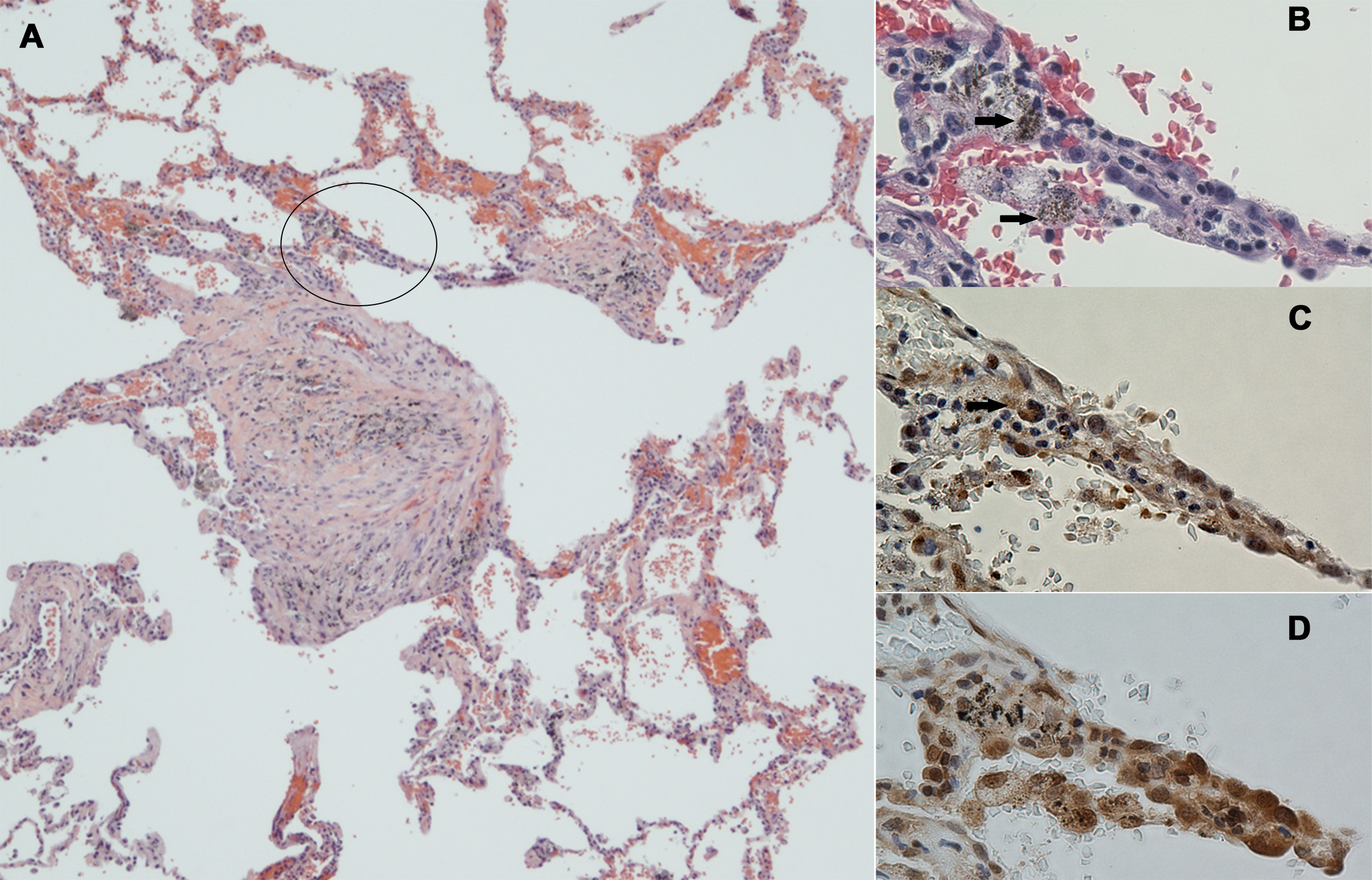

Supplement: Figure S2 — TNFα and NF-κB expression in silicosis. Panel A show photomicrograph (×100) illustrating hematoxyllin and eosin staining of silicotic nodule involving a terminal airway in lung tissue isolated from a silica exposed subject at the time of lung transplantation. Insert (circle) represents an area adjacent to the silicotic nodule to illustrate sequential HE (D ×200), and immunohistochemistry staining against TNFα in interstitial macrophages (arrows) loaded with dust (E ×200), or nuclear localization on NF-κB in adjacent epithelial cells (E ×200). (5.84 MB TIF) [file pone.0005689.s002.tif]

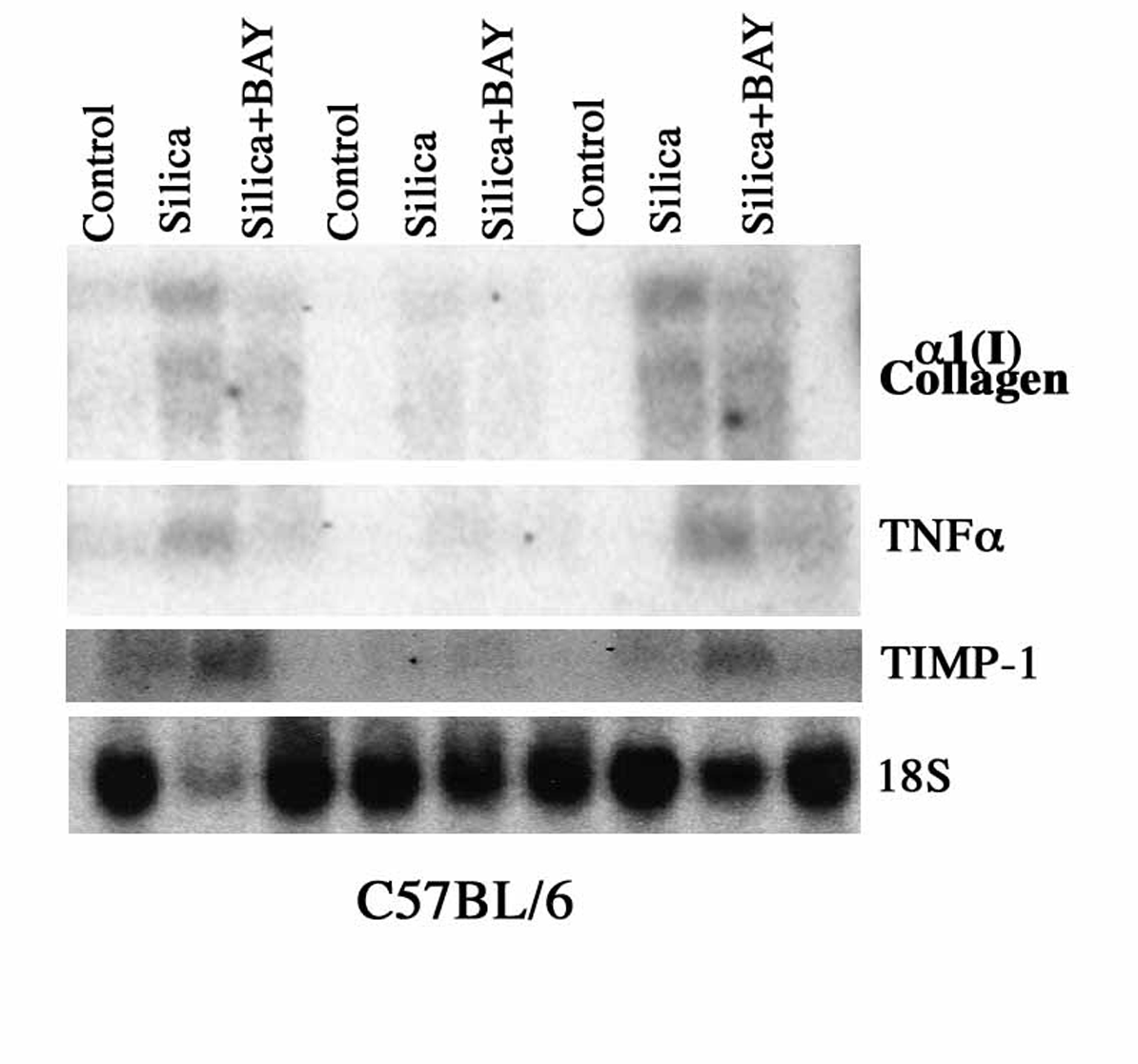

Supplement: Figure S3 — The Effect of BAY treatment on silica-induced gene expression in the mouse lung. Northern blot analysis of TNF, α1(I) collagen, TIMP-1, and 18S (loading control) mRNA expression in mouse lung 28 days following the intratracheal injection of saline as control, silica alone, or silica+BAY as described in Methods section. Gel is representative of result obtained with three different sets of animal exposures. (0.34 MB TIF) [file pone.0005689.s003.tif]

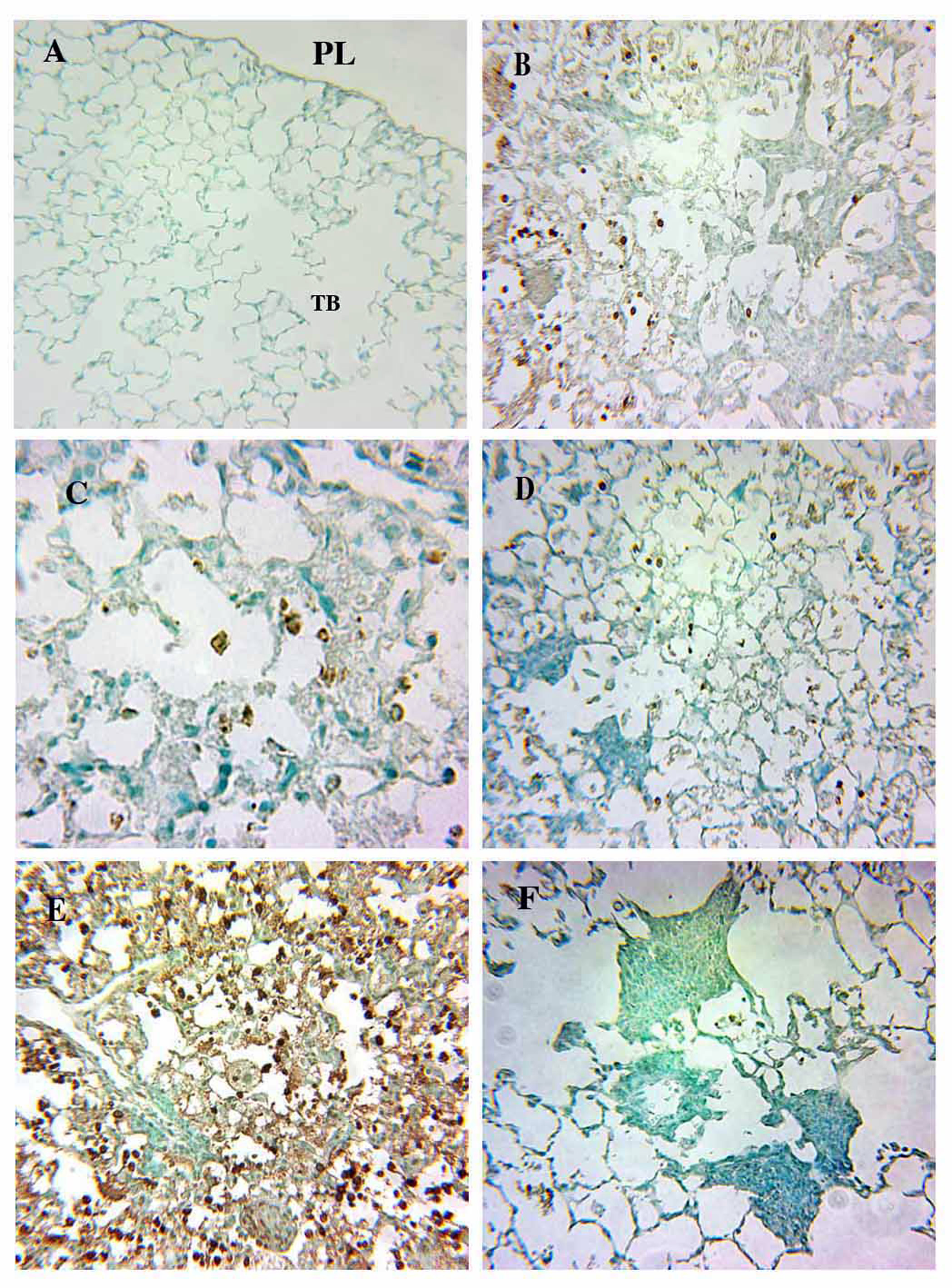

Supplement: Figure S4 — The effect of systemic or lung epithelial specific NF-κB inhibition on the induction of apoptosis (TUNEL staining) in the lung of silica exposed mice. Apoptosis was characterized by terminal deoxynucleotidyltransferase dUTP nick end-labeling (TUNEL) as described in Methods section. The panels show low (×100) power magnification photomicrographs of the lung obtained from lung tissues of C57BL/6 mice exposed to saline as control (A), silica (B), silica+BAY (D), or SPC-dnIκB transgenic mouse (E) exposed to silica as described in the Method section. Panel C show high (×400) magnification of TUNEL positive cells identified in B. Panel F illustrates negative staining (by omission of treatment with deoxynucleotidyltransferase enzyme) of a silica-exposed tissue used as control to demonstrate stain specificity. (10.28 MB TIF) [file pone.0005689.s004.tif]
