# Supplementary material for: Systemic Inhibition of NF-κB Activation Protects from Silicosis
Source: PLoS One. 2009 May 25;4(5):e5689. doi: 10.1371/journal.pone.0005689 (PMC2682759; doi:10.1371/journal.pone.0005689)
Supplement: Table S1 — (0.07 MB DOC) [file pone.0005689.s005.doc]

| **Symbol** | **Description** | **GeneBank** |  | |  |
| --- | --- | --- | --- | --- | --- |
|  |  |  |  | |  |
| Tnf | Tumor necrosis factor | NM_013693 |  | |  |
| Tnfrsf1b | Tumor necrosis factor receptor superfamily, member 1b | NM_011610 |  | |  |
| Ccl2 | Chemokine (C-C motif) ligand 2 | NM_011333 |  | |  |
| Tnfaip3 | Tumor necrosis factor, alpha-induced protein 3 | NM_009397 |  | |  |
| Il1b | Interleukin 1 beta | NM_008361 |  | |  |
| Relb | Avian reticuloendotheliosis viral (v-rel) oncogene related B | NM_009046 |  | |  |
| Csf2 | Colony stimulating factor 2 (granulocyte-macrophage) | NM_009969 |  | |  |
| Ikbkb | Inhibitor of kappaB kinase beta | NM_010546 |  | |  |
| Tnfrsf10b | Tumor necrosis factor receptor superfamily, member 10b | NM_020275 |  | |  |
| Csf3 | Colony stimulating factor 3 (granulocyte) | NM_009971 |  | |  |
| Cd27 | CD antigen 27 | NM_001033126 | |  | |
| Nfkb2 | Nuclear factor of kappa light polypeptide gene enhancer in B-cells 2, p49/p100 | NM_019408 |  | |  |
| Stat1 | Signal transducer and activator of transcription 1 | NM_009283 |  | |  |
| Ikbke | Inhibitor of kappaB kinase epsilon | NM_019777 |  | |  |
| Nfkb1 | Nuclear factor of kappa light chain gene enhancer in B-cells 1, p105 | NM_008689 |  | |  |
| Ifng | Interferon gamma | NM_008337 |  | |  |
| Tnfsf10 | Tumor necrosis factor (ligand) superfamily, member 10 | NM_009425 |  | |  |
| Rel | Reticuloendotheliosis oncogene | NM_009044 |  | |  |
| Ripk2 | Receptor (TNFRSF)-interacting serine-threonine kinase 2 | NM_138952 |  | |  |
| Irak2 | Interleukin-1 receptor-associated kinase 2 | NM_172161 |  | |  |
| Tnfrsf1a | Tumor necrosis factor receptor superfamily, member 1a | NM_011609 |  | |  |
| Nlrp12 | NLR family, pyrin domain containing 12 | XM_904112 |  | |  |
| Tnfsf14 | Tumor necrosis factor (ligand) superfamily, member 14 | NM_019418 |  | |  |
| Tbk1 | TANK-binding kinase 1 | NM_019786 |  | |  |
| Rela | V-rel reticuloendotheliosis viral oncogene homolog A (avian) | NM_009045 |  | |  |
| Cflar | CASP8 and FADD-like apoptosis regulator | NM_009805 |  | |  |
| Cd40 | CD40 antigen | NM_011611 |  | |  |
| Ripk1 | Receptor (TNFRSF)-interacting serine-threonine kinase 1 | NM_009068 |  | |  |
| Traf3 | Tnf receptor-associated factor 3 | NM_011632 |  | |  |
| Ikbkg | Inhibitor of kappaB kinase gamma | NM_010547 |  | |  |
| Ltbr | Lymphotoxin B receptor | NM_010736 |  | |  |
| Pcaf | P300/CBP-associated factor | NM_020005 |  | |  |
| Map3k1 | Mitogen-activated protein kinase kinase kinase 1 | NM_011945 |  | |  |
| Traf2 | Tnf receptor-associated factor 2 | NM_009422 |  | |  |
| Fadd | Fas (TNFRSF6)-associated via death domain | NM_010175 |  | |  |
| Irak1 | Interleukin-1 receptor-associated kinase 1 | NM_008363 |  | |  |
| Tradd | TNFRSF1A-associated via death domain | NM_001033161 |  | |  |
| Chuk | Conserved helix-loop-helix ubiquitous kinase | NM_007700 |  | |  |
| Egr1 | Early growth response 1 | NM_007913 |  | |  |
