# Supplementary material for: Systemic Inhibition of NF-κB Activation Protects from Silicosis
Source: PLoS One. 2009 May 25;4(5):e5689. doi: 10.1371/journal.pone.0005689 (PMC2682759; doi:10.1371/journal.pone.0005689)
Supplement: Methods S1 — File containing description of supplemental methods (0.05 MB DOC) [file pone.0005689.s006.doc]

***Generation of CCSP and SPC-dnIκB-α transgenic mice.*** The CCSP-dnIκB-α construct was generated by cloning a dominant-negative (dn) IκB mutant downstream of the airway epithelial-specific rat Clara cell secretory protein (CCSP) promoter (21). The dn mutant lacks the amino-terminal portion of the protein containing serine residues that are phosphorylated to target IκB for degradation (15). Initially a plasmid designated pCCSPA that contains the rat CCSP promoter fragment, a multiple cloning site, and an SV40 t antigen fragment with an intron and polyadenylation signal was generated as follows. A 0.5 kb SV40 fragment containing t antigen sequences was cloned into pBluescript II KS+ that had been cut with XbaI and SacI to generate pSVA. The 2.3 kb rat CCSP promoter fragment was isolated by PCR and cloned into Bluescript II KS+ cut with BamHI and SalI as described previously (21). This plasmid, designated pCCSP, was digested with BamHI, treated with the Klenow fragment of DNA polymerase I, and then digested with SalI to release the CCSP fragment. This fragment was inserted into pSVA that had been cut with XhoI, treated with the Klenow fragment of DNA polymerase I, and then digested with SalI. The resulting pCCSPA plasmid was cut with ClaI and Sma, and ligated with the 0.8 kb ClaI-SmaI dnIκB fragment from pCMV4∆NIκB (15) to produce pCCSP-dnIκB. A linear 3.7 kb fragment containing the CCSP-dnIκB construct free of vector sequences was isolated by digestion with Acc65I and SacII followed by gel purification. Transgenic mice carrying the CCSP-dnIκB construct were generated by microinjection of fertilized B6SJLF2 mouse eggs. Transgenic mice were genotyped by dot blot hybridization of tail biopsy DNA with a rat CCSP probe. Production of the dnIκB-α polypeptide in the transgenic mice was assessed by immunohistochemistry and Gel shift retardation assay of isolated Clara cells.

***CCSP-dnIκB DNA construct.*** The CCSP-dnIκB DNA construct contains a 2.3 kb fragment from the 5' region of the rat CCSP gene (gray), the entire translated portion (0.85 kb) of the dnIκB cDNA (white), and a 0.5 kb SV40 fragment containing an intron and polyadenylation signal (black).

SPC-dnIκBα transgenic mice were generated at the University of Washington and have been previously described (16). Lung tissue expression of the transgene was analyzed by Northern and Western blot, and gel shift retardation assay of isolated alveolar epithelial type II cells. CCSP and SPCdnIκBα mice were backcrossed a minimum of 7 times to C57BL/6 mice before they used in the silica challenge experiments. Transgene negative littermates were used as controls in all experiments. Mice were housed under SPF conditions in filtered cages, and permitted unlimited access to sterile food and water. The Animal Care Committees from the University of Pittsburgh and Tulane Health Science Center approved animal experiments.

***Isolation of Clara and Alveolar Epithelial type II (AEII) cells.*** Clara and AEII cells were isolated from the lung of transgenic and wild-type mice as previously described (24,25). Briefly, 20 ml of 0.9% NaCl followed by 3 ml of dispase (Sigma) were instilled intratracheally using a 3 ml syringe. The syringe was removed to dispel any excess dispase from the airways and then 0.45 ml of a 1% solution of low melting agarose (Sigma) was instilled into the lungs. The agarose was polymerized rapidly (2 min) by placing crushed ice over the lungs. The lung tissue was transferred to a 60 mm Petri dish containing 7 ml of DMEM supplemented with 0.01% type II DNase I (Sigma) and gently teased apart. The dissociated tissue was then passed successively through 100, 40, and 25 μM nylon mesh filters. Cells were recovered from the filtrate by centrifugation, suspended in DMEM supplemented with 10% fetal bovine serum, and plated onto 100 mm tissue culture dishes coated with rat anti mouse CD45 (42.5 μg) and rat anti mouse CD32 (16.5 μg) antibodies (PharMingen). Cells attached to the dishes were harvested and suspended at a concentration of 1x10^6^ cells/ml in Ham’s F12 medium supplemented with 15 mM HEPES. 2x10^6^ cells were used to prepare nuclear extracts for EMSA studies.

***cDNA Probes.*** cDNA templates used for experiments are as follows: the 1.101 kb murine TNF (pMuTNF) was obtained for American Type Culture Collection (Rockville, MD) and has been described elsewhere (12,20). The murine α1(I) procollagen plasmid (pMCollal-I), which contains a 321 bp insert from the *Eco*RI site near the translation stop codon to the *Hind*III site near the first stop codon was graciously provided by Dr. Eero Vuorio (Turku University, Turku, Finland). Mouse 18S [American Type Culture Collection (Rockville, MD)] cDNA was used for loading control (12,20).

***Northern Analysis and Ribonuclease Protected Assays.*** Total lung RNA was separated by electrophoresis (20 μg per lane) and transferred to Immobilon-N transfer membrane (Millipore, Bedford, MA). Membranes were hybridized with ^32^P-dCTP-labeled (ICN,Irvine, CA) random primed TNF, α1(I) procollagen, and 18S cDNAs described above. To evaluate signal density membranes were exposed to a Fuji phosphoimager (Fujix BAS 1000, Fuji USA, Standford, CT) plate overnight and scanned. Quantitative analysis was determined with the use of McBAS 2.5 software (Fuji USA). For each mRNA band the results were normalized to the internal control (18S) and expressed as a fold increase between the bands for the control and the bands for the silica-treated cells. Metalloproteinase and TIMP-1 RNA expression were evaluated using a RiboQuant^TM^ kit (Pharmingen) following the manufacturers recommendations

***Electrophoretic mobility Shift Assay (EMSA).*** Nuclear extracts from Clara cells, alveolar type II cells, or the lungs of silica-treated mice were prepared as previously described (23,26). For retardation assays, NF-κB consensus oligonucleotide 5'-GGGGACTTTCCC-3'- (Santa Cruz Biotechnology, Santa Cruz, CA) were end-labeled with [γ-^32^P] ATP and T4 polynucleotide kinase (GibcoBRL Life Technologies, Gaithersburgh, MD). 5 µg protein of crude nuclear extract will be mixed with the labeled probe and buffer in a 20 µl total volume and incubated to allow NF-κB binding to the probe. DNA-protein complexes were separated on 6% polyacrylamide gel (Novex, San Diego, CA). Gels were vacuum-dried and labeled complexes were detected by autoradiography. Competition assays were performed using 400 times excess of unlabeled probe or NF-κB mutant oligonucleotide (Santa Cruz). Supershifts were performed by adding to the binding mixture antibodies to p50 (sc1190X) or to p65 (sc372X) or against C-Jun/AP-1 (sc44X) (Santa Cruz) before adding the labeled probe to the mixture. Densitometric analysis of the gels was performed with the use of a Gel Doc 2000 (Bio-Rad).

***Western Blot Analysis.*** Lung tissues from control or silica exposed mice were collected as a function of time. 50 μg protein was separated on 10% SDS-PAGE and blotted onto nitrocellulose membranes (Schleicher & Schuell, Inc., Keene, NH). Membranes were incubated with an affinity purified rabbit polyclonal antibodies specific to phospho IκB or to total IκB from the PhosphoPlus IκB -alpha (Ser^32^) antibody kit (New England Biolabs) in 5% milk in TBS-T. Blots were incubated with the appropriate peroxidase-conjugated secondary antibody and developed using an enhanced chemiluminescence detection kit (ECL, Amersham Life Science, Inc., Piscataway, NJ).
